# Association between Serum Kalirin Levels and the *KALRN* gene rs9289231 Polymorphism in Early-Onset Coronary Artery Disease 

**Published:** 2018-04

**Authors:** Afsaneh Shafiei, Younes Pilehvar-Soltanahmadi, Shayan Ziaee, Mohsen Mofarrah, Nosratollah Zarghami

**Affiliations:** 1 *Department of Biochemistry, Payame Noor University of Tehran, Tehran, Iran.*; 2 *Department of Medical Biotechnology, Faculty of Advanced Medical Sciences, Tabriz University of Medical Sciences, Tabriz, Iran.*; 3 *Tehran Heart Center, Tehran Unversity of Medical Sciences, Tehran, Iran.*

**Keywords:** *KALRN protein, human*, *Polymorphism, single nucleotide*, *Genotype*, *Coronary artery disease*

## Abstract

**Background:** Recently, rs9289231 genetic variations of kalirin (KALRN) have been introduced as potential genetic markers for coronary artery disease (CAD). However, the influence of KALRN single-nucleotide polymorphisms (SNPs) on serum kalirin levels has not been investigated in CAD patients so far. Thus, the present study aimed to survey whether SNP T>G (rs9289231) was associated with the risk of early-onset CAD and serum kalirin levels among the study subjects.

**Methods:** The rs9289231 polymorphism of the KALRN was genotyped in 512 subjects (61.5% male, mean age=46.3±7.1 y), comprising 268 subjects with angiographically diagnosed CAD and 244 controls using an HRM assay. Also, the levels of serum kalirin were compared between 133 CAD subjects and 123 controls using a sandwich ELISA assay.

**Results:** The CAD subjects had more frequently GG genotypes than the controls. The odds ratio (OR) remained significant after adjustment for known CAD risk factors (OR=4.13, 95% CI: 2.48–9.10; P<0.001). A significant difference was also observed in that the G allele was more frequent among the CAD subjects. The G allele at the rs9289231 polymorphism was associated with a higher risk of CAD (OR=2.11, 95% CI: 1.27–2.59; P=0.001). The mean kalirin level of the CAD patients was higher than that of the controls (P=0.041). No significant correlation was seen in the different genotypes with serum kalirin levels.

**Conclusion:** The KALRN rs9289231 T>G variant was considerably related with an increased risk of early-onset CAD. High kalirin levels were found in young CAD patients compared to the control subjects, with the levels not affected by the different genotypes of rs9289231.

## Introduction

Coronary artery disease (CAD) is the most widespread type of cardiovascular diseases. Universally, CAD is the principal cause of mortality and is expected to remain so for the next 20 years. Annually, approximately 7.2 million men and women die due to CAD. In 2020, it is predicted that this disease will be responsible for a total of 11.1 million deaths worldwide.^1^^-^^3^ 

CAD is identified as a complex multifactorial disorder and many classical risk factors such as diet, hypertension, age, smoking, hyperlipidemia, and diabetes mellitus can affect it.^   4 ^ In addition, molecular interactions between genetic variations and environmental factors play a major role in the progression of CAD, particularly in young immature patients.^   5 ^ As is the case with other intricate disorders, the innate susceptibility of CAD can be attributed to several genes. Identification of individuals who are at risk of developing CAD without current symptoms is still considered the primary prevention strategy of coronary heart disease in many countries.^6^^, ^^7^ Genome-wide linkage analysis is an unbiased approach that can identify unknown genes and genetic susceptibility loci associated with CAD. For example, loci on 9q21 and 3q13 were mentioned more frequently among previous studies due to the remarkable correlation between their genetic variations and vascular pathologies such as CAD, stroke, and atherosclerosis.^8^^, ^^9^ 

At the chromosome 9q CAD risk locus, *GATA2* (MIM 137295) and *KALRN* (MIM 604605) have been reported as 2 candidate genes associated with CAD.^   10 ^ Wang and colleagues during an in-depth investigation of the 3q chromosome found that an intronic single-nucleotide polymorphism (SNP) rs9289231 of the *KALRN* gene was related to early-onset CAD. Furthermore, the risk allele of this SNP was involved in atherosclerosis burden.^11^^, ^^12^

*KALRN* is a large gene located in the 3q21 chromosomal region; it encodes a protein called “kalirin”. Kalirin is a guanine nucleotide exchange factor (GEF) with widespread cell signaling functions which activate Rac1, RhoA, and RhoG.^   13 ^ The different kalirin isoforms have been shown to play an important role in spine morphogenesis, longitudinal bone growth, cortical morphology, and smooth-muscle-cell signaling.^   14^

To our knowledge, there have been only a few studies published regarding the SNPs of *KALRN* and their association with CAD. Moreover, the influence of *KALRN* SNPs on serum kalirin levels has yet to be investigated in CAD patients.

The aim of the present study was to investigate whether there is a relationship between the polymorphism gene *KALRN* (rs9289231) and the risk of early-onset CAD and also serum kalirin levels.

## Methods

In this cross-sectional study, subjects were recruited from a consecutive sample of 512 young individuals, consisting of 268 CAD subjects and 244 controls, who were undergoing coronary angiography for the confirmation of suspected myocardial ischemia and the evaluation of CAD at Tehran Heart Center, a hospital affiliated with Tehran University of Medical Sciences in Iran. Candidates were selected from a pool of individuals referred for angiography between April 2007 and March 2009, and age criteria for early-onset CAD were defined as before 45 years for men and 55 years for woman. Hypertension was deﬁned as a systolic blood pressure higher than 140 mm Hg and a diastolic blood pressure higher than 90 mm Hg. Diabetes mellitus was confirmed when the subject had hemoglobin A1c (HbA1c) levels equal to or greater than 6.5% and/or a fasting plasma glucose concentration higher than 126 mg/dL with signs of diabetes mellitus. All the participants signed an informed written consent, and the study was approved by the Ethics Committee of Tehran Heart Center. Medical history and relevant lifestyle aspects information were obtained by interviewing the candidates.

A venous blood sample (10 mL) was collected from all the individuals after an overnight fast (12 to 14 h)*. *Blood cell fraction, serum, and plasma were frozen at -20 ^°^C. For all the subjects, the concentrations of serum glucose, cholesterol, triglyceride, and high-density lipoprotein cholesterol were measured via enzymatic and colorimetric methods (Roche COBAS INTEGRA 400 plus AutoAnalyzer). In total, 256 random samples of the study subjects (50% of the total, power=0.82) were assayed for the serum levels of kalirin using commercially available sandwich ELISA kits (CUSABIO BIOTECH CO., Ltd., Wuhan, China). The detection range was 23.44 to 1500 pg/mL. The minimum detectable dose of human kalirin is typically less than 5.86 pg/mL. The sensitivity of this assay or the lower limit of detection was defined as the lowest protein concentration that could be differentiated from 0 and intra-assay precision (precision within an assay) was CV% less than 8%. The body mass index (BMI) was calculated as weight (kg)/(height)^2 ^(m)^2^ ratios.

Isolation of deoxyribonucleic acid (DNA) was performed with 8 mL of whole blood according to a modified National Institutes of Health protocol (a standard salting-out method) using lysis buffer and DNA extraction reagents.^   15 ^ DNA quality and quantity were assessed through the optical density ratio (OD260/OD280) and the Beer–Lambert law by UV spectrophotometry. The high resolution melt (HRM) assay is a platform for the exposure of mutations that can be used to recognize small differences in DNA sequences by assessing changes in the shape of their melting curve indices compared to standard (wild-type) DNA.

**Figure1:**
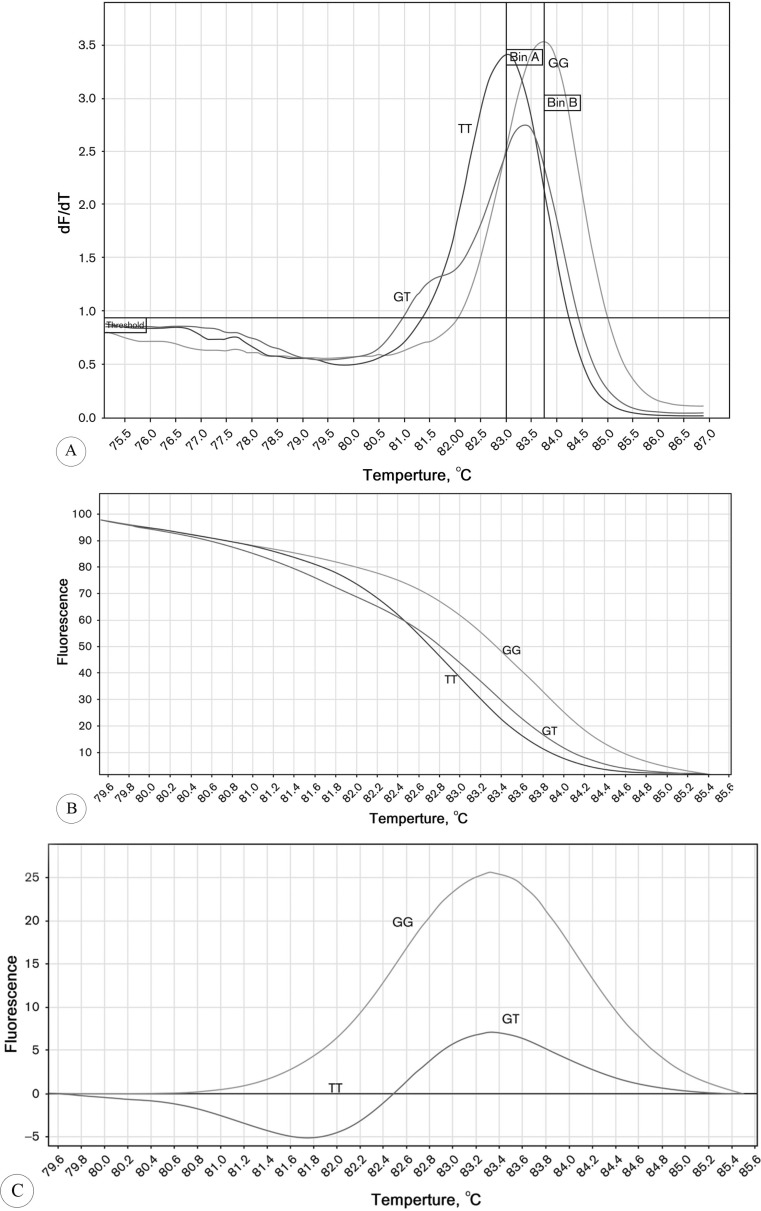
High-resolution melt (HRM) analysis melting curves.

SNP genotyping was carried out using an HRM assay on a Rotor-Gene 6000 real-time analyzer (Corbett Life Science Pty. Ltd., Mortlake, Australia). The rs9289231 *KALRN *variant among wild type, heterozygous, and homozygous mutant melting profiles was discriminated. As is shown in Figure 1, alterations in fluorescence through a gradient temperature procedure reveal different patterns of DNA melting. We designed 2 specific primers according to the common HRM requirements, comprising a forward primer, GCATCCCTCCAGCAGTCAG, and a reverse primer, CCAACAGATTTTGTAATGTATCAC. The final reaction mixture (20 µL per reaction) included Type-it HRM Master Mix (QIAGEN NV, Venlo, Netherlands) with accommodating DNA-binding dye (EvaGreen [Biotium Inc., Hayward, CA]), DNA polymerase (5U/mL), primer mix (0.4 µM of each primer), genomic DNA (50 ng), and RNase Free Water (QIAGEN NV). The polymerase chain reaction (PCR) product length was 142 bp. We used 6 DNA samples after sequencing and detecting SNP rs9289231 genotype as the controls to obtain the confidence percent of SNP genotyping by the HRM procedures. DNA sequence analysis was performed using an ABI PRISM 3100 genetic analyzer. In each batch of the experiment, negative controls (no template controls) were analyzed too.

The continuous variables between the 2 groups were analyzed by parametric or nonparametric methods, depending on the normality of their distribution as determined by the Kolmogorov–Smirnov test. Logistic regression was performed to calculate the odds ratio (OR) (95% confidence intervals [CIs]) of the genotypes between the case and control groups. Log-transformed concentrations geometric mean was calculated for the serum levels of kalirin. A p value less than 0.05 was considered statistically significant. All the statistical assessments were performed using the SPSS, version 18 (SPSS Inc., Chicago, IL). The allele frequencies of the polymorphism were analyzed using the χ^2 ^test according to the Hardy-Weinberg equilibrium law.

A) Derivative data plots (-dF/dT vs. temperature): The melt curves peaked at the maximum dissociation rate on the basis of the T_m_.

B) Normalized DNA melting curves: Comparison of the 3 genotype patterns was made possible by the normalization of these plots.

C) Differentiation curves: This graph displays the difference between each sample and a given genotype control and allows a calculated percentage confidence relative to a known genotype.

dF/dT, Derivative of fluorescence over temperature ; TT, T allele carriers; GT, G and T alleles carriers; GG, G allele carrier

Peak bins (A, B, and C), Bins are used to define the general area where we expect peaks to occur. The peaks of the curves close together were assigned to the closest bin.

## Results

The baseline anthropometric and clinical parameters of the patients and the controls are displayed in Table 1. As was expected, the patients revealed high values of different cardiovascular risk factors in comparison with the control group. There were no statistically significant differences in age, BMI, and family history of CAD (P=0.102, 0.241, and 0.300, respectively). 

SNP rs9289231 was successfully genotyped in the *KALRN* gene. The genotype frequencies in the control population were in the Hardy–Weinberg equilibrium (χ^2^=1.37), but the case group exhibited a deviation on the basis of the Hardy–Weinberg proportion (χ^2^=27.1). The results revealed a different genotype distribution between the patient and control groups in this study. A total of 291 (56.8%) subjects had TT, 156 (30.5%) had GT, and 65 (12.7%) had GG genotypes. The CAD subjects more frequently had the GG genotype and less frequently the TT genotype than the controls (19.5% vs. 5.3% and 49.6% vs. 64.7%, respectively) (Table 2). The OR for CAD was 4.13 (95% CI: 2.48-9.10; P<0.001) of the GG genotype after adjustment for hypertension, diabetes, hyperlipidemias, and current smoking determined as confounding risk factors. A signiﬁcant difference was also observed insofar as the G allele was more frequent among the CAD subjects (34% vs. 21%). The G allele at the rs9289231 polymorphism was associated with a higher risk of CAD (OR=2.11, 95% CI: 1.27-2.59; P=0.001) (Table 2).

The concentrations of serum kalirin were determined in 256 subjects (133 CAD patients and 123 control subjects). The geometric mean kalirin level of the CAD patients was 120.25±106.70 pg/mL, which was higher than that of the control subjects (76.37±52.42 pg/mL) (P=0.041) (Table 1).

The different genotypes of rs9289231 of the *KALRN* gene were not signiﬁcantly associated with the levels of the serum kalirin in the case and control groups (Table 3). 

**Table 1:** Anthropometric and clinical characteristic parameters of the early-onset CAD and control subjects*

	Normal Coronary Subjects (n=244)	Early-Onset CAD Patients (n=268)	P
Age (y)	46.1±6.3	46.5±8.0	0.102
Male gender	124 (51.1)	191 (71.3)	<0.001
Body mass index (kg/m^2^)	28.5±4.4	29.2±5.2	0.241
Hypertension	86 (35.4)	121 (45)	0.028
Hyperlipidemias	143 (58.7)	199 (74.4)	<0.001
Diabetes mellitus	47 (19.1)	84 (31.4)	0.002
Current smoking	32 (13.1)	97 (36.1)	<0.001
Family history of CAD	70 (28.7)	88 (32.9)	0.300

CAD, Coronary artery disease

* Data are presented as geometric mean±SD or n (%).

**Table 2:** SNP rs9289231 Genotype prevalence and allele frequency in the case and control individuals*

*KALRN* Genotypes/Alleles	Normal Coronary Subjects (n=244)	Early-Onset CAD Patients (n=268)	Odd Ratio	95% CI	P
TT	158 (64.7)	133 (49.6)	Reference		
GT	73 (30.0)	83 (30.9)	1.35	0.91–1.99	0.131
GG	13 (5.3)	52 (19.5)	4.13	2.48–9.10	<0.001
T allele	389 (79)	349 (65)	Reference		
G allele	99 (21)	187 (35)	2.11	1.27–2.59	0.001

SNP, Single nucleotide polymorphism; CAD, Coronary artery disease; GG, Homozygous carrier of a G allele; GT, Heterozygous carrier of G and T alleles; TT, Homozygous carrier of a T allele

* Data are presented as n (%)

**Table 3:** Levels of serum kalirin according to the genotype of rs9289231*

rs9289231	Normal Coronary Subjects (n=123)		Early-Onset CAD Patients (n=133)		P
	n	kalirin (pg/mL)	n	kalirin (pg/mL)	
Alleles					0.079
TT	78	45.30±28.38	64	72.46±39.56	
GT	35	75.33±36.75	39	157.10±131.69	
GG	10	99.38±70.87	30	108.48±93.61	
Total	123	76.37±52.42	133	120.25±106.70	0.041

CAD, Coronary artery disease; TT, Homozygous carrier of a T allele; GT, Heterozygous carrier of G and T alleles; GG, Homozygous carrier of a G allele

* Data are presented as geometric mean± approximate SD

## Discussion

The findings of our study revealed a signiﬁcant difference in the kalirin serum levels between the CAD and control groups in that the CAD patients had higher kalirin levels than the controls. We also found that the different genotypes of rs9289231 at the *KALRN* gene were significantly associated with the risk of early-onset CAD but not with the levels of serum kalirin among the Iranian population.

The roles of *KALRN* in the development of atherosclerosis are not evidently characterized in the literature. Kalirin, as a GEF, catalyzes nucleotide exchange for Rac-1 and other guanosine-5'-triphosphate (GTP)-binding proteins such as Rho A. Rac-1 is a key factor in smooth muscle cell (SMC) signaling and motility in response to extracellular signals via the regulation of nicotinamide adenine dinucleotide phosphate (NADPH) oxidases. It is possible that the activation of Rho GTPase by kalirin is involved in the cell signaling of Rac-1 and Rho A, which can regulate SMC adhesion, proliferation, and migration and also actin organization through the stimulation of a downstream signaling pathway.^13^^, ^^16^^, ^^17^ 

Based on these clarifications, there are several hypotheses that genetic variations in *KALRN* result in endothelial dysfunction and atherosclerosis. The first hypothesis is that kalirin promotes SMC migration and proliferation by influencing the Rac-1 signaling pathway and platelet-derived growth factor (PDGF) receptor-β and G protein-coupled receptors. Wu et al.^   18 ^ showed that loss of kalirin function reduced SMC migration principally by reducing Rac1 activation in a loss-of-function mouse model. Kalirin can mediate SMC migration through the downstream of multiple receptor tyrosine kinases such as EphB2 and its agonist ephrinB2 in SMCs and monocyte/macrophages.^18^^, ^^19^ Wu et al.^   20 ^ suggested that the EphB2–kalirin signaling axis could promote monocyte/macrophage infiltration and contribute significantly to neointimal hyperplasia. On the basis of another hypothesis, kalirin may also contribute to neointimal hyperplasia through interaction with the N-terminal domain of NOS2 and thereby inhibit nitric oxide synthase (NOS2) activity and reduce SMC proliferation and mitochondrial respiration.

Previous studies have demonstrated that different levels of kalirin in SMCs, endothelial cells, and monocytes lead to different functions in injured arteries; consequently, the serum kalirin level might be an early marker for preatherosclerotic intimal hyperplasia.

Our study supports the hypothesis that *KALRN* is a CAD susceptibility gene. Although our results indicated a significant association between certain *KALRN* polymorphisms and CAD, it is also possible that other associated genes such as *CdGap* and *MYLK* may be involved in a common Rho GTPase-signaling pathway with potential interacting functions that progress CAD.^11^^, ^^21^ 

The first study on the association between kalirin and CAD was conducted by Wang and colleagues,^11^ who concluded that kalirin had an important role in the Rho GTPase signaling pathway and that rs9289231 SNP had the strongest association with CAD. Further, Boroumand et al.^   22 ^ demonstrated an association between the G allele of rs9289231 polymorphism of kalirin and an increased risk of CAD.

Despite a few works on the association between the *KALRN* gene polymorphism and the risk of CAD, the current literature has a dearth of data on serum kalirin levels in CAD patients. We sought to investigate whether the rs9289231 polymorphism of the *KALRN* gene was associated with the risk of CAD and serum kalirin levels in an Iranian population.

In the present study, the frequencies of the GG genotype and the G allele of rs9289231 in the CAD patients were higher than those of the control group and not only the GG genotype but also the G allele of rs9289231 was correlated with an increased risk of CAD without affecting the serum kalirin level. This finding may be explained by the fact that rs9289231 is located in intron and might not have affected the final serum kalirin levels. In addition, limitations in the sample size of the present study may in part explain this issue. 

## Conclusion

The *KALRN* gene rs9289231 T>G variant was linked to an increased risk of CAD among our study subjects. Additionally, our CAD patients had higher levels of serum kalirin, with the levels not affected by the different genotypes of rs9289231. Enlarging the sample size and performing further replication studies would make the conclusions more convincing.

## Notes:

***This paper should be cited as: ***Shafiei A, Pilehvar-Soltanahmadi Y, Ziaee S, Mofarrah M, Zarghami N. Association between Serum Kalirin Levels and the KALRN gene rs9289231 Polymorphism in Early-Onset Coronary Artery Disease. J Teh Univ Heart Ctr 2018;13(2)58-64.
